# Increased Light Exposure Alleviates One Form of Photoreceptor Degeneration Marked by Elevated Calcium in the Dark

**DOI:** 10.1371/journal.pone.0008438

**Published:** 2009-12-24

**Authors:** Xiaoqing Liu, Basil S. Pawlyk, Michael Adamian, Elena V. Olshevskaya, Alexander M. Dizhoor, Clint L. Makino, Tiansen Li

**Affiliations:** 1 Berman-Gund Laboratory for the Study of Retinal Degenerations, Harvard Medical School, Massachusetts Eye & Ear Infirmary, Boston, Massachusetts, United States of America; 2 Hafter Research Laboratories, Pennsylvania College of Optometry, Salus University, Elkins Park, Pennsylvania, United States of America; 3 Howe Laboratory, Harvard Medical School, Massachusetts Eye & Ear Infirmary, Boston, Massachusetts, United States of America; 4 Neurobiology, Neurodegeneration and Repair Laboratory, National Eye Institute, Bethesda, Maryland, United States of America; Duke University, United States of America

## Abstract

**Background:**

In one group of gene mutations that cause photoreceptor degeneration in human patients, guanylyl cyclase is overactive in the dark. The ensuing excess opening of cGMP-gated cation channels causes intracellular calcium to rise to toxic levels. The Y99C mutation in guanylate cyclase-activating protein 1 (GCAP1) has been shown to act this way. We determined whether prolonged light exposure, which lowers cGMP levels through activation of phototransduction, might protect photoreceptors in a line of transgenic mice carrying the GCAP1-Y99C.

**Methodology/Principal Findings:**

We reared cohorts of GCAP1-Y99C transgenic mice under standard cyclic, constant dark and constant light conditions. Mouse eyes were analyzed by histology and by immunofluorescence for GFAP upregulation, a non-specific marker for photoreceptor degeneration. Full-field electroretinograms (ERGs) were recorded to assess retinal function. Consistent with our hypothesis, constant darkness accelerated disease, while continuous lighting arrested photoreceptor degeneration.

**Conclusions/Significance:**

In contrast to most forms of retinal degeneration, which are exacerbated by increased exposure to ambient light, a subset with mutations that cause overly active guanylyl cyclase and high intracellular calcium benefitted from prolonged light exposure. These findings may have therapeutic implications for patients with these types of genetic defects.

## Introduction

Over-exposure to light, either in terms of intensity or duration, generally exerts a deleterious effect on retinal photoreceptors with underlying genetic mutations. Conversely, light deprivation often exhibits a sparing effect compared to standard cyclic lighting conditions [1], [2], [3], [4], [5], [6], [7], [8]. For example, prolonged light exposure accelerated photoreceptor degeneration in transgenic mice carrying mutant forms of rhodopsin and in mice lacking rhodopsin kinase or arrestin. In contrast, photoreceptor degeneration was milder in these lines of mice kept under constant darkness. In situations where the underlying mutations lead to unregulated activation of the phototransduction cascade, a beneficial effect of reduced environmental light would be easily understood. Indeed, patients with hereditary photoreceptor degeneration are advised to reduce light exposure as a possible ameliorative therapy for their condition.

Photoreceptors sense light through a signaling cascade known as phototransduction. Light isomerizes rhodopsin, leading to the sequential activation of transducin and phosphodiesterase (PDE6). PDE6 hydrolyzes cGMP resulting in closure of cGMP-gated cation channels located in the plasma membrane of the outer segments. As a result, calcium influx ceases upon illumination and intracellular Ca^2+^ decreases. Guanylyl cyclases (GCs) synthesize and replenish cGMP. Retinal GCs in vertebrates are subject to regulation through guanylate cyclase-activating proteins 1 and 2 (GCAP1 and 2), EF-hand calcium/magnesium-binding proteins that activate GCs at lower Ca^2+^ in the light but inhibit GCs at higher Ca^2+^ in the dark [9], [10], [11], [12]. One of the GCAPS, GCAP1, has been implicated in retinal degenerative diseases. Certain mutant alleles of GCAP1, for example Y99C and I143NT, lower the calcium binding affinity of GCAP1 [13], [14]. As a result, over-stimulation of GCs leads to abnormally high levels of free cGMP and intracellular Ca^2+^ in the dark. The cytotoxicity of high Ca^2+^ influx has been extensively documented in numerous systems[15], including photoreceptors[16]. This is the likely mechanism by which GCAP1 mutations cause dominantly inherited photoreceptor degeneration in humans[13], [14] and in transgenic mice[17].

In the GCAP1-Y99C transgenic mice, photoreceptors degenerate under standard cyclic lighting[17]. Pathologically high levels of intracellular Ca^2+^ manifest only in darkness[17] because in the light, activated PDE effectively eliminates free cGMP and permits Ca^2+^ to fall to the normal minimum. In this way, phototransduction could in theory override the deleterious effect of this mutant. Hence we predicted that photoreceptors were vulnerable to insults incurred by the GCAP1-Y99C mutation only in the dark-adapted state and that shortening of the dark-adapted state would promote photoreceptor survival. We tested this hypothesis in the Y99C transgenic mice and report that increased light exposure successfully preserved their photoreceptors for as long as 10 months.

## Materials and Methods

### Animals

A line of transgenic mice (L52H; in C57Bl/6 background) carrying the Y99C mutation in GCAP1 was previously described[17]. The L52H line expresses the mutant protein at a level similar to the endogenous GCAP1 expression and undergoes photoreceptor degeneration at a moderate rate when reared under standard cyclic light conditions[17]. In the present study, the L52H mice were raised under cyclic lighting until they were approximately 3 weeks of age (age of weaning). Each litter was then divided into two groups. One group (n = 15) was kept under constant dark and the other group (n = 15) was kept under constant light (100–200 lux; slightly dimmer than typical indoor room lighting). After three to ten months in constant light or constant dark, mice were analyzed by ERG, histological analysis and immunostaining. All transgenic mice enrolled in the study had their genotype verified by PCR. Since a murine rhodopsin promoter was placed upstream of the transgene, the L52H line was genotyped by PCR with one primer matching the murine rhodopsin promoter and the other primer matching the GCAP1 gene (5′-CTGGGATTTCCATGGCTGAGGTG and 5′-TCAACCCGCAGCCTCCGCTGCCAGGTC). Wild-type (WT) C57Bl/6 mice, shown for comparison in this study, and additional L52H transgenic mice were also reared under cyclic lighting. All experiments adhered to the ARVO Statement for the Use of Animals in Ophthalmic and Vision Research and were approved by the Institutional Animal Care and Use Committee.

### Immunofluorescence and Histology

Enucleated eyes were fixed with 2% formaldehyde in 0.1 M phosphate buffered saline (PBS) (pH 7.2) and the anterior segments were removed. After soaking in 30% sucrose, the eyecups were shock frozen in liquid nitrogen. Sections were cut at a thickness of 10 µm and incubated with GFAP monoclonal antibody (G-3893) (Sigma) at a 1∶500 dilution for two hours at room temperature. After washing three times in PBS, sections were incubated for 1–2 hours with Alexa Fluor®546 conjugated goat anti-mouse IgG (Molecular Probes, Oregon, USA) at a 1∶500 dilution. For histological analysis, enucleated eyes were placed in 1% formaldehyde and 2.5% glutaraldehyde in 0.1 M sodium cacodylate buffer (pH 7.4) and the anterior segments and lens were removed. After fixation overnight at 4°C, the tissues were washed with PBS. The tissues were post-fixed with 2% osmium tetroxide in PBS for 2 hours at room temperature and then washed twice with distilled water. After dehydration with a graded series of ethanol, the tissues were embedded in Epon resin. Sections were cut along the vertical meridian through the optic nerve head, at a thickness of 1 µm and stained with 1% sodium borate consisting of equal portions of 2% Azure II and 2% methylene Blue. For morphometric analyses of photoreceptor inner and outer segment (IS/OS) length and outer nuclear layer (ONL) thickness, measurements were made along the vertical meridian (superior to inferior) at 3 locations to each side of the optic nerve head separated by about 500 µm each. Measurements began at about 500 µm from the optic nerve head itself.

### Electroretinogram Recording

Electroretinograms (ERGs) were recorded as described[18], [19]. Briefly, following an overnight dark adaptation mice were anesthetized with sodium pentobarbital at 80 mg/kg given intraperitoneally. Their pupils were dilated with 0.2% phenylephrine and 0.02% cyclopentolate hydrochloride. Full-field, rod-dominant (>95%) ERGs were elicited with 10 µsec flashes of white light (4.3 log ft-Lt) presented at 1-minute intervals in a Ganzfeld dome.

### Statistical Analysis

Differences in mean ERG amplitude, ONL thickness and combined inner/outer segment lengths between dark and light rearing groups were evaluated by the Student *t*-test.

## Results and Discussion

**Figure 1:**
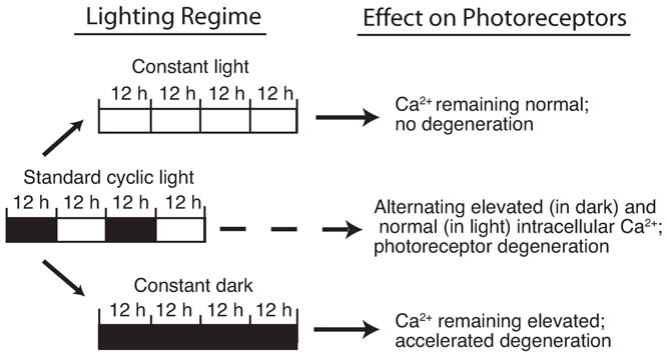
Model for the disease mechanism in the GCAP1 mutant mice and the impact of light exposure. Intracellular calcium is elevated in the dark, but falls to the normal minimum in the light. Hence the dark state presents a pathogenic condition but the light state does not. Prolongation of the light phase at the expense of the dark phase is predicted to enhance photoreceptor survival.

Our central hypothesis was that photoreceptors were subjected to the pathogenic insult of the GCAP1-Y99C mutation only in the dark. We therefore predicted that any prolongation of the light phase at the expense of the dark phase in a lighting cycle would favor photoreceptor survival (Fig. 1). As a test of this hypothesis, we studied cohorts of GCAP1-Y99C (L52H line) transgenic mice (GCAP1 mice) reared under different lighting conditions. GCAP1 mice succumb to a moderate rate of photoreceptor degeneration under standard cyclic lighting conditions with severe shortening of inner and outer segments and loss of more than half of all photoreceptors by 6 months of age [17].

**Figure 2:**
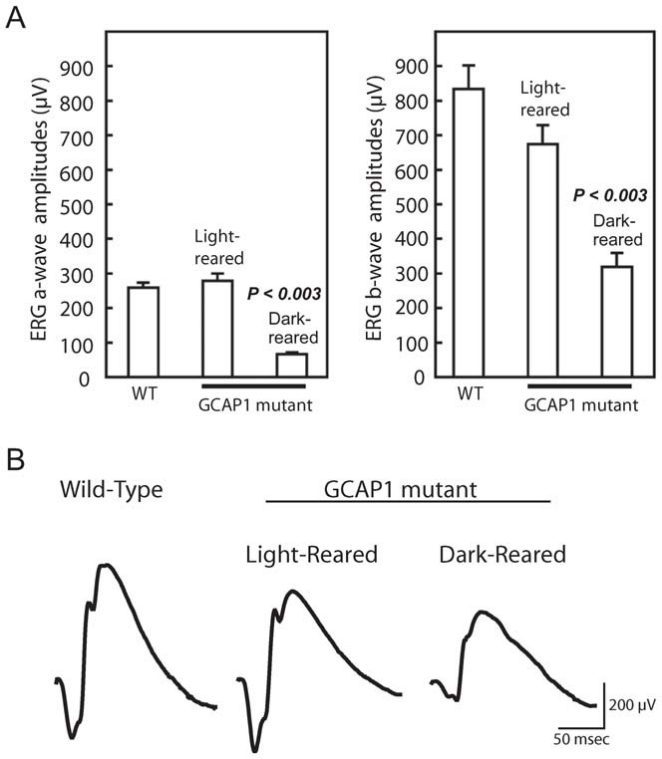
Preservation of retinal function by constant light. (A) The L52H line of mutant mice were raised from birth under cyclic lighting until 3 weeks of age, when they were switched to constant light or constant darkness and kept for an additional three months. Dark-adapted, full-field ERGs with 10 µsec flashes of white light (4.3 log ft-L) were elicited in a Ganzfeld dome. ERG a- and b-waves represent the activities of photoreceptors and inner retinal neurons, respectively. In mutant mice kept under constant light, the a-wave amplitude of 271±29 µV (mean±SEM) was well within the range for age-matched cyclic light reared WT mice (258±15 µV), while the b-wave amplitude of 654±68 µV was slightly below the WT average of 837±68 µV (n = 6 each). Under dark-rearing conditions, the a- and b-wave amplitudes declined to 72±6 µV and 318±40 µV (n = 5), respectively. The ERG a- and b-wave amplitudes in mice kept in constant light were significantly higher than those kept in the dark (*P<0.003*). (B) Representative dark-adapted, rod dominant ERG waveforms recorded from GCAP1 mutant mice reared in dark or light for 3 months, as well as waveforms from an age matched WT mouse.

To control for any effects of litter, GCAP1 mice were first raised under dim cyclic lighting from birth until weaning at 3 weeks of age. The litter was then divided and placed in either constant dark or constant light. Three months later, we found by ERG recordings a severe deficit in retinal function in mice reared in the dark but nearly normal retinal function in those kept in the light (Fig. 2A). Mean dark-adapted (rod dominated) ERG a- and b-wave amplitudes for mutant mice kept in constant lighting (n = 6) were 271 µV and 654 µV, respectively. These values are similar to or just below those recorded from WT mice (n = 6) of the same age (258 µV and 837 µV for a- and b-wave amplitudes, respectively). In contrast, mutant mice kept in the dark (n = 5) for this period had ERG a- and b wave amplitudes (72 µV and 318 µV, respectively) that were significantly below those of both WT and light-kept mutant mice (*P<0.003*). Representative ERG waveforms from dark and light kept mutant mice as well as from a WT are shown in Fig. 2B.

**Figure 3:**
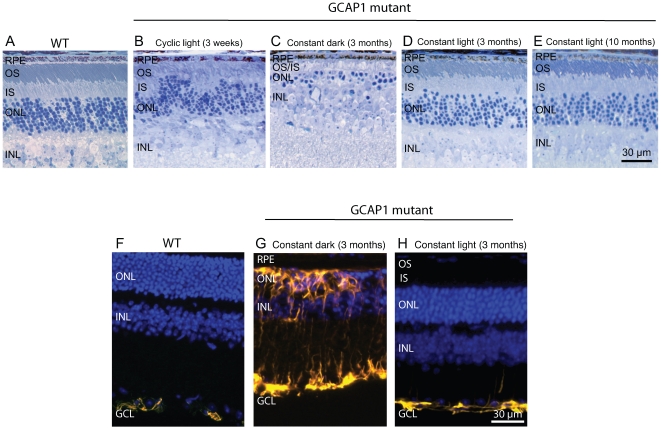
Preservation of photoreceptor cells in the GCAP1 mutant mice by constant light. (A) Wild-type mouse (B) Degeneration had already begun in GCAP1 mutant mice raised from birth under standard cyclic lighting for only 3 weeks of age as indicated by the presence of pyknotic nuclei as well as disorganized and shortened outer segments (n = 3). (C) After three months of constant darkness only approx. 2 rows of photoreceptors remained in mutant mice, and the inner and outer segments were severely shortened. (D) In contrast, mutant mice kept in constant light showed no further loss of photoreceptors and outer segment organization actually improved, as compared to the earlier time when they initially entered the constant light condition. (E) Even after 10 months in constant light, photoreceptors continued to show excellent preservation (n = 3). (F) Wild-type retina staining for GFAP shows the typical, very mild signal restricted to the GCL. (H) Similar to wild-type retina, only mild GFAP immunostaining was seen in mutant retina after three months under constant light whereas widespread GFAP immunostaining in the dark-reared mutant retina (G) was consistent with severe photoreceptor degeneration. GFAP was stained yellow. Cell nuclei were stained blue with Hoechst dye 33342. RPE, retinal pigment epithelium; OS, outer segments; IS, inner segments; ONL, outer (photoreceptor) nuclear layer; INL, inner nuclear layer; GCL, ganglion cell layer.

**Figure 4:**
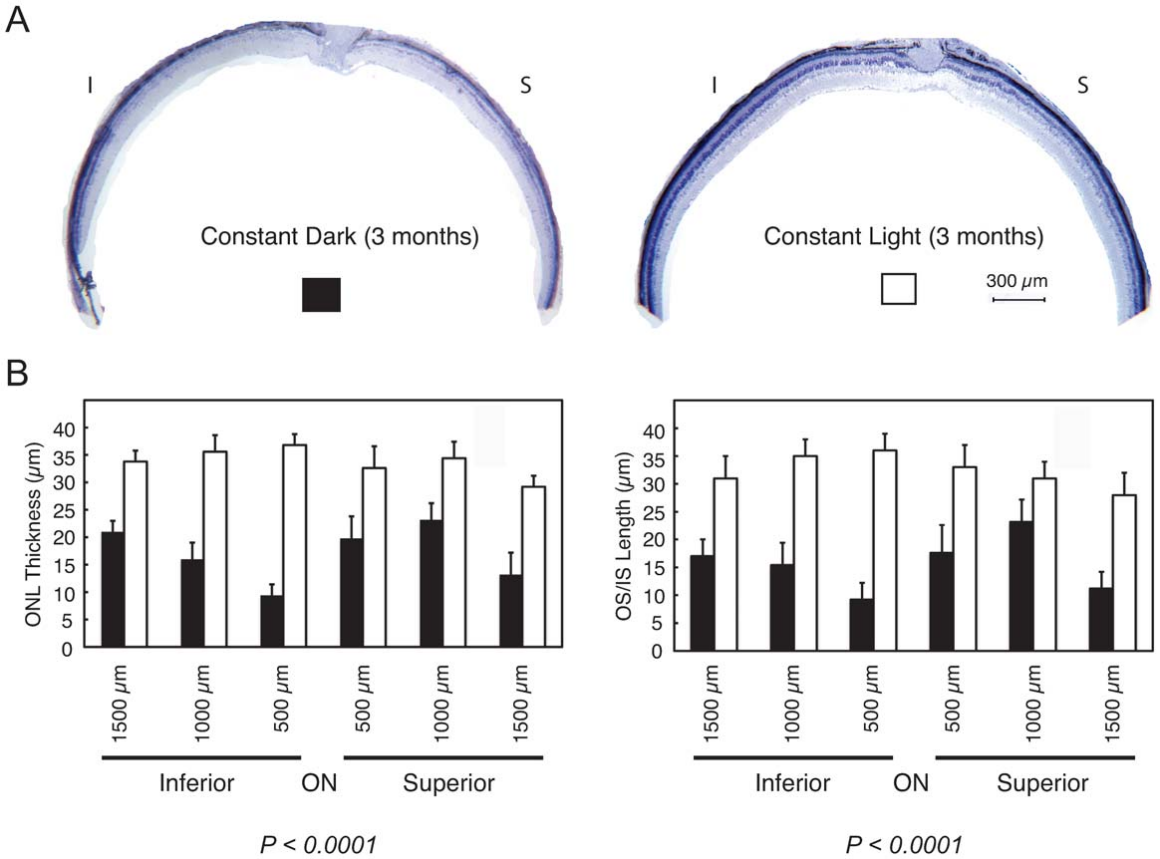
Morphometric analyses of mutant eyes after 3 months in constant darkness or constant light. A. Light micrographs of inferior (I) and superior (S) hemispheres of a representative pair of dark- and light-reared mutant retinas. B. Rescue of photoreceptor cells in different regions of the mutant retina. Shown are morphometric measurements of outer nuclear layer thickness (left) and combined inner and outer segment lengths (right) along the vertical meridian from dark and light reared mutant retinas (mean±SEM; n = 6 each group, *P<0.0001* for both parameters).

Histological analysis showed that the mutant retinas at 3 weeks of age under cyclic lighting already had visible ongoing photoreceptor degeneration. This is indicated by the appearance of pyknotic nuclei, and shortened and disorganized outer segments (Fig. 3B). Three months of constant darkness drove the progression of photoreceptor degeneration to near completion with only about 2 rows of cells remaining at this time, with severely shortened inner segments and no discernable outer segments (Fig. 3C). This is a faster rate of degeneration than that seen in mice kept under the standard cyclic lighting condition which retained 4–5 rows of photoreceptor nuclei and shortened inner and outer segments (data not shown). In contrast, mutant animals kept under constant light during this period showed substantially better morphology, with 6–7 rows of photoreceptor cell nuclei and well organized inner and outer segments (Fig. 3D). The photoreceptor layer kept under constant light for three months was slightly thinned compared to an age and background-matched WT mouse retina (Fig. 3A), which may be accounted for by cell loss which had occurred prior to the change in lighting condition. The well-organized outer segments in mice kept in constant light suggest a degree of repair to this dynamic structure (compare Fig. 3D to 3B). The sparing effect of constant light was long lasting, such that mice analyzed 10 months after switching to constant light (n = 3) showed little progression of degeneration in the photoreceptor cell layer (Fig. 3E). In contrast, photoreceptor degeneration in mice of the same line kept under standard cyclic lighting had progressed to completion by 7 months, with approximately a single layer of cells remaining (n = 6; data not shown). We also found that glial fibrillary acidic protein (GFAP), a sensitive marker for retinal gliosis in response to photoreceptor degeneration[19], was strongly upregulated in mice kept in the dark but not in mice kept in the light (Fig. 3F–H). Figure 4B shows morphometric analysis of retinal histology from a group of mutant mice raised in cyclic light for 3 weeks after birth and then switched to either constant darkness or constant light for 3 months. The combined inner and outer segment length and outer nuclear layer thickness were measured at three locations on either side of the optic nerve head along the vertical meridian of the retinal sections. Measurements from three independent locations from each hemisphere were combined and analyzed. The inner and outer segments were significantly longer in mutant eyes kept in constant light (32.3 µm) than in eyes kept in constant darkness (15.5 µm) (n = 6 each group, *P<0.0001*). The outer nuclear layer was also considerably thicker in mutant eyes kept in constant light (33.8 µm) than in eyes kept in constant darkness (17.0 µm) (n = 6 each group, *P<0.0001*).

Both photoreceptor function and morphology in mutant mice reared in constant light for 3 months approached wild-type mouse levels. The less than perfect rescue in these light-reared mice could be attributed to at least two factors. The first is that mice were placed under constant light at about 3 weeks of age when photoreceptor loss had already begun as manifested by the appearance of pyknotic nuclei. The second is that although the lighting environment remained constant, the experimental condition likely achieved only prolongation of the light phase in a light/dark cycle rather than continuous illumination at the level of the retina. This is because the mice were darkly pigmented and they tended to huddle when sleeping or resting, creating undefined “dark” periods in terms of actual light reaching the retina. A genetically engineered, built-in “equivalent light”, based on manipulation of the phototransduction cascade, would ensure continuous pseudo light stimulation. Recently, when a faster degenerating line that over-expresses the GCAP1-Y99C mutant (L53) was crossed with a constitutively active rhodopsin mutant (rhodopsin G90D), there was a strong suppression of photoreceptor degeneration [20]. This observation suggests that the “equivalent light” from a constitutively active rhodopsin mutant largely offset the effect of the GCAP1 mutation. The effect of constant light was also explored in that study in the L53 line of mutant mice; a protective effect was indicated although to a much lesser extent than that of the G90D transgene. The present study is a more comprehensive evaluation of the effect of ambient light on photoreceptor degeneration in this type of mutant, which shows that the protective effect of ambient light can be very strong in 52H mice where the levels of Y99C GCAP1 are not as high as they were in the L53 mice[17]. Our findings not only support the conclusions of the previous study done with a different mouse model, but also directly show that constant light quite efficiently rescues photoreceptor degeneration when the Ca^2+^ -insensitive GCAP1 mutant is expressed at the levels similar to that of the wild type GCAP1, whereas constant dark exacerbates the disease.

Because the GCAP1-Y99C mutant was controlled by a rhodopsin promoter in the transgenic mice and thus would not drive proper expression in cones, we limited our assays in this study to rod photoreceptors. Clinical studies show that the Y99C mutation in GCAP1 causes primarily a cone dystrophy (with involvement of both rods and cones) in humans [21]. Based on the generally similar principles of phototransduction regulation in rods and cones, it can be reasonably argued that the GCAP1-Y99C mutant may damage cone photoreceptors through a similar mechanism. Therefore cone photoreceptor degeneration caused by GCAP1-Y99C mutant are predicted to also respond favorably to prolonged light exposure.

Hereditary photoreceptor degeneration is genetically heterogeneous. Our study shows that light avoidance may not always be beneficial in photoreceptor degenerative disease and, in some instances, may cause more harm to photoreceptors. How mutant photoreceptors respond to light/dark regimes will differ depending on specific disease mechanisms. For a class of gene mutations that result in elevated intracellular calcium in the dark, minimizing the dark state may alleviate the disease (Fig. 1). This class of mutations includes but is not limited to the GCAP1 gene. Several dominant alleles in human retGC1 gene (GUCY2D) are believed to result in similar overactivity of GC1 in the dark and elevated intracellular calcium[22]. Certain mutations in CNGA3 and CNGB3 give rise to cyclic nucleotide gated channels that are hypersensitive to cGMP and probably raise intracellular calcium with normal levels of guanylyl cyclase activity[23] [24], [25]. Our data suggest that increased light exposure may be explored as a potential therapeutic strategy for patients with this type of photoreceptor degeneration. We hypothesize that the lighting regime described here could be readily adapted into everyday life, e.g., sleeping with a dim light on, with minimal untoward effect.
